# 1,2-Bis(1,3-benzothia­zol-2-yl)benzene

**DOI:** 10.1107/S1600536808042542

**Published:** 2008-12-20

**Authors:** Guo-wei Wang, Lin-ping Wu, Ling-hua Zhuang, Jin-tang Wang

**Affiliations:** a Department of Light Chemical Engineering, College of Science, Nanjing University of Technology, Nanjing 210009, People’s Republic of China; b Department of Applied Chemistry, College of Science, Nanjing University of Technology, Nanjing 210009, People’s Republic of China

## Abstract

The title compound, C_20_H_12_N_2_S_2_, was prepared by the reaction of *o*-phthalic acid and 2-amino­thio­phenol under microwave irradation. The phenyl ring, *A*, and the benzothia­zolyl rings, *B* and *C*, are planar; the dihedral angles are *A*/*B* = 19.9 (11), *A*/*C* = 87.8 (3) and *B*/*C* = 84.4 (4)°. Weak inter­molecular C—H⋯N hydrogen bonds link the mol­ecule, forming zigzag chains parallel to the *c* axis.

## Related literature

For details of the synthesis and applications of benzothia­zoles, see: Chakraborti *et al.* (2004 ▶); Seijas *et al.* (2007 ▶). For the use of microwave-assisted organic synthesis, see: Kappe & Stadler (2005 ▶). For bond-length data, see: Allen *et al.* (1987 ▶).
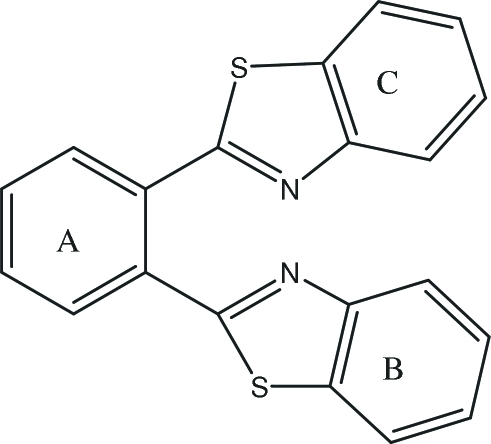


## Experimental

### 

#### Crystal data

C_20_H_12_N_2_S_2_
*M* *_r_* = 344.44
Monoclinic, 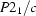

*a* = 10.748 (2) Å
*b* = 19.148 (4) Å
*c* = 8.1840 (16) Å
β = 100.77 (3)°
*V* = 1654.6 (6) Å^3^
*Z* = 4
Mo *K*α radiation
μ = 0.32 mm^−1^
*T* = 293 (2) K
0.30 × 0.20 × 0.10 mm

#### Data collection

Enraf–Nonius CAD-4 diffractometer
Absorption correction: ψ scan (North *et al.*, 1968 ▶) *T*_min_ = 0.909, *T*_max_ = 0.968
3000 measured reflections
3000 independent reflections
1640 reflections with *I* > 2σ(*I*)
3 standard reflections every 200 reflections intensity decay: 9%

#### Refinement

*R*[*F* ^2^ > 2σ(*F* ^2^)] = 0.069
*wR*(*F* ^2^) = 0.206
*S* = 1.10
3000 reflections
217 parameters
H-atom parameters constrained
Δρ_max_ = 0.31 e Å^−3^
Δρ_min_ = −0.32 e Å^−3^

### 

Data collection: *CAD-4 Software* (Enraf–Nonius, 1989 ▶); cell refinement: *CAD-4 Software*; data reduction: *XCAD4* (Harms & Wocadlo, 1995 ▶); program(s) used to solve structure: *SHELXS97* (Sheldrick, 2008 ▶); program(s) used to refine structure: *SHELXL97* (Sheldrick, 2008 ▶); molecular graphics: *ORTEPIII* (Burnett & Johnson, 1996 ▶), *ORTEP-3 for Windows* (Farrugia, 1997 ▶) and *PLATON* (Spek, 2003 ▶); software used to prepare material for publication: *SHELXL97*.

## Supplementary Material

Crystal structure: contains datablocks global, I. DOI: 10.1107/S1600536808042542/dn2416sup1.cif

Structure factors: contains datablocks I. DOI: 10.1107/S1600536808042542/dn2416Isup2.hkl

Additional supplementary materials:  crystallographic information; 3D view; checkCIF report

## Figures and Tables

**Table 1:** Hydrogen-bond geometry (Å, °)

*D*—H⋯*A*	*D*—H	H⋯*A*	*D*⋯*A*	*D*—H⋯*A*
C12—H12⋯N2^i^	0.93	2.46	3.370 (7)	165

Symmetry code: (i) 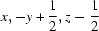.

**Fig. 1.**
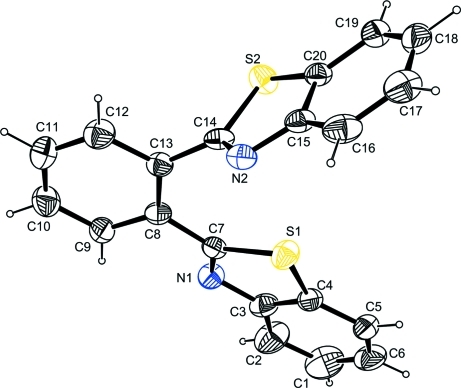
A view of the molecular structure of (I) with the atom-numbering scheme. Ellipsoids are drawn at the 50% probability level. H atoms are represented as small spheres of arbitrary radii.

**Fig. 2.**
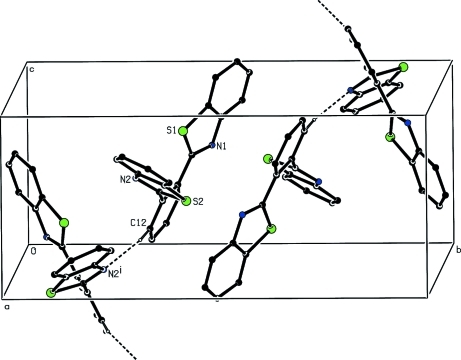
Partial packing view of (I) showing the C-H···N hydrogen bonds shown as dashed lines. [Symmetry code: (i) x, -y+1/2, z-1/2 ]

## supplementary crystallographic information

### Comment

Benzothiazole are remarkable heterocyclic ring systems. They have been found to exhibit a wide spectrum of biological activities. They have shown antitumor,antimalarial,and fungicide activity. They are also an important class of industrial chemicals. Many kinds of 2-substituted benzothiazoles are utilized as vulcanization accelators in the manufacture of rubber,as fluorescent brightening agents in textile dyeing,and in the leather industry (Chakraborti *et al.*,2004; Seijas *et al.*,2007). There are numerous synthetic methods to produce 2-arylbenzothiazoles. The most important ones include the reaction of *o*-aminothiophenols with benzoic acids or their derivatives (Chakraborti *et al.*,2004; Seijas *et al.*,2007). Microwave-assisted organic synthesis (MAOS) is a powerful technique that is being used more and more to accelerate thermal organic reactions (Kappe & Stadler, 2005). We are focusing on Microwave-assisted synthesis of new products of bisbenzothiazole. We here report the crystal structure of the title compound (I).

The phenyl ring A (C8/C9/C13), benzothiazolyl ring B(C1/C2/C6/C7) and benzothiazolyl ring C(C14/C15/C20) are planar (Fig. 1). The dihedral angles between them are A/B = 19.9°, A/C = 87.8°, B/C = 84.4°, respectively. All bond lengths are within normal ranges (Allen *et al.*, 1987). There are weak intermolecular C—H···N hydrogen bonds whick link the molecule forming zig-zag chains parallel to the c axis .(Table 1, Fig.2).

### Experimental

A mixture of 2-aminothiophenol (2.5 g, 20 mmol), 5 ml orthophosphoric acid, 5 g polyphosphoric acid and *o*-phthalic acid (1.66 g, 10 mmol) in a beakerflask (150 ml) was placed in a domestic microwave oven (0.8 KW, 2450 MHz) and irradiated (micromode, full power) for 4 min(30 s per time). The reaction mixture was cooled to r.t. and washed with aq NaOH (20%, 150 ml), The pH was adjusted to 10, the resulted solide was filtered. Then the crude compound(I) was obtained. It was crystallized from ethanol. Crystals of (I) suitable for X-ray diffraction were obtained by slow evaporation of methanol. ^1^H NMR (DMSO, δ, p.p.m.) 7.35–7.40 (m, 2 H), 7.46–7.51 (m, 2 H), 7.64 (dd,2 H), 7.81 (d, 2 H), 7.95 (dd,2 H), 8.05 (d,2 H).

### Refinement

All H atoms were positioned geometrically, with C—H = 0.96 and 0.97 Å for methyl and methylene H atoms, and constrained to ride on their parent atoms, with *U*_iso_(H) = *xU*_eq_(C), where *x*= 1.5 for methyl H and *x* = 1.2 for methylene H atoms.

### Crystal data

**Table:**

C_20_H_12_N_2_S_2_	*F*(000) = 712
*M**_r_* = 344.44	*D*_x_ = 1.383 Mg m^−^^3^
Monoclinic, *P*2_1_/*c*	Mo *K*α radiation, λ = 0.71073 Å
Hall symbol: -P 2ybc	Cell parameters from 27 reflections
*a* = 10.748 (2) Å	θ = 1–25°
*b* = 19.148 (4) Å	µ = 0.32 mm^−^^1^
*c* = 8.1840 (16) Å	*T* = 293 K
β = 100.77 (3)°	Block, yellow
*V* = 1654.6 (6) Å^3^	0.30 × 0.20 × 0.10 mm
*Z* = 4	

### Data collection

**Table:**

Enraf–Nonius CAD-4 diffractometer	1640 reflections with *I* > 2σ(*I*)
Radiation source: fine-focus sealed tube	*R*_int_ = 0.0000
graphite	θ_max_ = 25.3°, θ_min_ = 1.9°
ω/2θ scans	*h* = −12→12
Absorption correction: ψ scan (North *et al.*, 1968)	*k* = 0→22
*T*_min_ = 0.909, *T*_max_ = 0.968	*l* = 0→9
3000 measured reflections	3 standard reflections every 200 reflections
3000 independent reflections	intensity decay: 9%

### Refinement

**Table:**

Refinement on *F*^2^	Primary atom site location: structure-invariant direct methods
Least-squares matrix: full	Secondary atom site location: difference Fourier map
*R*[*F*^2^ > 2σ(*F*^2^)] = 0.069	Hydrogen site location: inferred from neighbouring sites
*wR*(*F*^2^) = 0.206	H-atom parameters constrained
*S* = 1.10	*w* = 1/[σ^2^(*F*_o_^2^) + (0.0773*P*)^2^ + 1.3256*P*] where *P* = (*F*_o_^2^ + 2*F*_c_^2^)/3
3000 reflections	(Δ/σ)_max_ < 0.001
217 parameters	Δρ_max_ = 0.31 e Å^−^^3^
0 restraints	Δρ_min_ = −0.32 e Å^−^^3^

### Special details

**Table:**

Geometry. All esds (except the esd in the dihedral angle between two l.s. planes) are estimated using the full covariance matrix. The cell esds are taken into account individually in the estimation of esds in distances, angles and torsion angles; correlations between esds in cell parameters are only used when they are defined by crystal symmetry. An approximate (isotropic) treatment of cell esds is used for estimating esds involving l.s. planes.
Refinement. Refinement of *F*^2^ against ALL reflections. The weighted *R*-factor *wR* and goodness of fit *S* are based on *F*^2^, conventional *R*-factors *R* are based on *F*, with *F* set to zero for negative *F*^2^. The threshold expression of *F*^2^ > σ(*F*^2^) is used only for calculating *R*-factors(gt) *etc*. and is not relevant to the choice of reflections for refinement. *R*-factors based on *F*^2^ are statistically about twice as large as those based on *F*, and *R*- factors based on ALL data will be even larger.

### Fractional atomic coordinates and isotropic or equivalent isotropic displacement parameters (Å^2^)

**Table:**

	*x*	*y*	*z*	*U*_iso_*/*U*_eq_	
C1	0.1724 (9)	0.5336 (4)	0.9897 (12)	0.108 (3)	
H1	0.1202	0.5630	1.0376	0.130*	
C2	0.1344 (7)	0.5137 (4)	0.8270 (9)	0.096 (2)	
H2	0.0583	0.5291	0.7640	0.116*	
C3	0.2149 (6)	0.4693 (3)	0.7600 (7)	0.0610 (15)	
C4	0.3256 (5)	0.4465 (3)	0.8557 (6)	0.0524 (13)	
C5	0.3633 (6)	0.4661 (3)	1.0209 (7)	0.0664 (16)	
H5	0.4376	0.4496	1.0862	0.080*	
C6	0.2832 (8)	0.5121 (4)	1.0827 (9)	0.085 (2)	
H6	0.3064	0.5284	1.1911	0.102*	
C7	0.2792 (4)	0.4036 (3)	0.5708 (6)	0.0456 (12)	
C8	0.2677 (5)	0.3692 (3)	0.4050 (6)	0.0457 (12)	
C9	0.1489 (5)	0.3646 (3)	0.3069 (7)	0.0586 (15)	
H9	0.0800	0.3835	0.3453	0.070*	
C10	0.1297 (6)	0.3325 (3)	0.1532 (8)	0.0712 (18)	
H10	0.0485	0.3300	0.0897	0.085*	
C11	0.2287 (6)	0.3044 (3)	0.0938 (8)	0.0702 (17)	
H11	0.2159	0.2832	−0.0102	0.084*	
C12	0.3461 (6)	0.3079 (3)	0.1882 (8)	0.0664 (16)	
H12	0.4133	0.2888	0.1464	0.080*	
C13	0.3715 (5)	0.3394 (3)	0.3479 (6)	0.0474 (12)	
C14	0.5036 (5)	0.3402 (3)	0.4373 (6)	0.0486 (13)	
C15	0.6821 (5)	0.3021 (3)	0.5897 (6)	0.0478 (13)	
C16	0.7649 (6)	0.2577 (3)	0.6947 (8)	0.0701 (17)	
H16	0.7366	0.2153	0.7297	0.084*	
C17	0.8887 (6)	0.2782 (4)	0.7449 (8)	0.0744 (18)	
H17	0.9447	0.2498	0.8157	0.089*	
C18	0.9307 (6)	0.3412 (4)	0.6907 (7)	0.0682 (17)	
H18	1.0155	0.3532	0.7240	0.082*	
C19	0.8532 (5)	0.3858 (3)	0.5915 (7)	0.0590 (15)	
H19	0.8835	0.4280	0.5583	0.071*	
C20	0.7256 (4)	0.3666 (3)	0.5396 (6)	0.0470 (12)	
N1	0.1893 (4)	0.4454 (2)	0.5978 (5)	0.0545 (12)	
N2	0.5565 (4)	0.2888 (2)	0.5286 (5)	0.0538 (11)	
S1	0.40034 (14)	0.39075 (8)	0.73836 (18)	0.0629 (5)	
S2	0.60337 (13)	0.40998 (7)	0.41671 (19)	0.0594 (5)	

### Atomic displacement parameters (Å^2^)

**Table:**

	*U*^11^	*U*^22^	*U*^33^	*U*^12^	*U*^13^	*U*^23^
C1	0.126 (7)	0.104 (7)	0.109 (7)	0.032 (6)	0.056 (6)	−0.017 (5)
C2	0.102 (6)	0.112 (6)	0.083 (5)	0.036 (5)	0.038 (4)	−0.013 (5)
C3	0.071 (4)	0.061 (4)	0.058 (4)	0.003 (3)	0.029 (3)	−0.004 (3)
C4	0.058 (3)	0.054 (3)	0.048 (3)	−0.007 (3)	0.017 (3)	0.000 (3)
C5	0.077 (4)	0.075 (4)	0.048 (3)	−0.020 (3)	0.014 (3)	−0.005 (3)
C6	0.114 (6)	0.085 (5)	0.067 (4)	−0.017 (5)	0.048 (4)	−0.024 (4)
C7	0.038 (3)	0.048 (3)	0.051 (3)	−0.001 (2)	0.010 (2)	0.008 (2)
C8	0.049 (3)	0.041 (3)	0.050 (3)	0.003 (2)	0.016 (2)	0.005 (2)
C9	0.047 (3)	0.073 (4)	0.055 (4)	−0.002 (3)	0.010 (3)	−0.006 (3)
C10	0.057 (4)	0.083 (5)	0.069 (4)	0.000 (3)	−0.001 (3)	0.005 (4)
C11	0.079 (4)	0.068 (4)	0.061 (4)	−0.002 (4)	0.007 (3)	−0.013 (3)
C12	0.072 (4)	0.060 (4)	0.071 (4)	0.007 (3)	0.023 (3)	−0.010 (3)
C13	0.050 (3)	0.042 (3)	0.053 (3)	0.000 (2)	0.017 (2)	−0.003 (2)
C14	0.049 (3)	0.051 (3)	0.052 (3)	0.007 (2)	0.026 (2)	0.004 (3)
C15	0.047 (3)	0.055 (3)	0.045 (3)	0.013 (2)	0.019 (2)	0.009 (2)
C16	0.076 (4)	0.069 (4)	0.072 (4)	0.015 (3)	0.030 (3)	0.023 (3)
C17	0.075 (4)	0.082 (5)	0.069 (4)	0.022 (4)	0.021 (4)	0.013 (4)
C18	0.056 (3)	0.094 (5)	0.055 (4)	0.007 (3)	0.011 (3)	−0.010 (4)
C19	0.059 (3)	0.065 (4)	0.055 (3)	−0.003 (3)	0.016 (3)	−0.005 (3)
C20	0.043 (3)	0.056 (3)	0.045 (3)	0.006 (2)	0.015 (2)	0.005 (2)
N1	0.045 (2)	0.062 (3)	0.057 (3)	0.015 (2)	0.012 (2)	0.000 (2)
N2	0.053 (3)	0.054 (3)	0.058 (3)	−0.002 (2)	0.021 (2)	0.002 (2)
S1	0.0625 (9)	0.0730 (11)	0.0537 (9)	0.0142 (8)	0.0116 (7)	−0.0059 (8)
S2	0.0572 (9)	0.0493 (8)	0.0699 (10)	−0.0033 (7)	0.0072 (7)	0.0119 (7)

### Geometric parameters (Å, °)

**Table:**

C1—C6	1.352 (10)	C10—H10	0.9300
C1—C2	1.372 (10)	C11—C12	1.352 (8)
C1—H1	0.9300	C11—H11	0.9300
C2—C3	1.395 (8)	C12—C13	1.418 (7)
C2—H2	0.9300	C12—H12	0.9300
C3—C4	1.369 (7)	C13—C14	1.471 (7)
C3—N1	1.383 (7)	C14—N2	1.301 (6)
C4—C5	1.389 (7)	C14—S2	1.740 (5)
C4—S1	1.730 (5)	C15—N2	1.373 (6)
C5—C6	1.391 (9)	C15—C16	1.402 (7)
C5—H5	0.9300	C15—C20	1.409 (7)
C6—H6	0.9300	C16—C17	1.374 (8)
C7—N1	1.305 (6)	C16—H16	0.9300
C7—C8	1.492 (7)	C17—C18	1.390 (9)
C7—S1	1.723 (5)	C17—H17	0.9300
C8—C9	1.379 (7)	C18—C19	1.352 (8)
C8—C13	1.409 (7)	C18—H18	0.9300
C9—C10	1.381 (8)	C19—C20	1.407 (7)
C9—H9	0.9300	C19—H19	0.9300
C10—C11	1.360 (8)	C20—S2	1.712 (5)
			
C6—C1—C2	122.1 (7)	C10—C11—H11	120.5
C6—C1—H1	118.9	C11—C12—C13	123.1 (6)
C2—C1—H1	118.9	C11—C12—H12	118.4
C1—C2—C3	117.2 (7)	C13—C12—H12	118.4
C1—C2—H2	121.4	C8—C13—C12	116.7 (5)
C3—C2—H2	121.4	C8—C13—C14	125.5 (5)
C4—C3—N1	116.0 (5)	C12—C13—C14	117.8 (5)
C4—C3—C2	120.4 (6)	N2—C14—C13	123.7 (5)
N1—C3—C2	123.6 (6)	N2—C14—S2	115.2 (4)
C3—C4—C5	122.3 (6)	C13—C14—S2	121.0 (4)
C3—C4—S1	108.9 (4)	N2—C15—C16	125.4 (5)
C5—C4—S1	128.8 (5)	N2—C15—C20	114.4 (4)
C4—C5—C6	115.9 (6)	C16—C15—C20	120.2 (5)
C4—C5—H5	122.0	C17—C16—C15	118.5 (6)
C6—C5—H5	122.0	C17—C16—H16	120.7
C1—C6—C5	121.9 (7)	C15—C16—H16	120.7
C1—C6—H6	119.0	C16—C17—C18	120.5 (6)
C5—C6—H6	119.0	C16—C17—H17	119.8
N1—C7—C8	119.1 (4)	C18—C17—H17	119.8
N1—C7—S1	115.2 (4)	C19—C18—C17	122.6 (6)
C8—C7—S1	125.6 (4)	C19—C18—H18	118.7
C9—C8—C13	119.0 (5)	C17—C18—H18	118.7
C9—C8—C7	117.9 (4)	C18—C19—C20	118.2 (6)
C13—C8—C7	123.0 (4)	C18—C19—H19	120.9
C8—C9—C10	121.6 (5)	C20—C19—H19	120.9
C8—C9—H9	119.2	C19—C20—C15	119.9 (5)
C10—C9—H9	119.2	C19—C20—S2	130.4 (4)
C11—C10—C9	120.5 (6)	C15—C20—S2	109.7 (4)
C11—C10—H10	119.7	C7—N1—C3	110.2 (4)
C9—C10—H10	119.7	C14—N2—C15	111.4 (4)
C12—C11—C10	119.0 (6)	C7—S1—C4	89.6 (3)
C12—C11—H11	120.5	C20—S2—C14	89.4 (3)

### Hydrogen-bond geometry (Å, °)

**Table:**

*D*—H···*A*	*D*—H	H···*A*	*D*···*A*	*D*—H···*A*
C12—H12···N2^i^	0.93	2.46	3.370 (7)	165

Symmetry codes: (i) *x*, −*y*+1/2, *z*−1/2.
